# A** machine learning approach to seizure detection in a rat model of post-traumatic epilepsy**

**DOI:** 10.1038/s41598-023-40628-1

**Published:** 2023-09-22

**Authors:** Robert J. Kotloski

**Affiliations:** 1 Department of Neurology, William S Middleton Memorial Veterans Hospital, Madison, WI 53705 USA; 2 Department of Neurology, University of Wisconsin School of Medicine and Public Health, 1685 Highland Avenue, Madison, WI 53705-2281 USA

**Keywords:** Epilepsy, Learning algorithms

## Abstract

Epilepsy is a common neurologic condition frequently investigated using rodent models, with seizures identified by electroencephalography (EEG). Given technological advances, large datasets of EEG are widespread and amenable to machine learning approaches for identification of seizures. While such approaches have been explored for human EEGs, machine learning approaches to identifying seizures in rodent EEG are limited. We utilized a predesigned deep convolutional neural network (DCNN), GoogLeNet, to classify images for seizure identification. Training images were generated through multiplexing spectral content (scalograms), kurtosis, and entropy for two-second EEG segments. Over 2200 h of EEG data were scored for the presence of seizures, with 95.6% of seizures identified by the DCNN and a false positive rate of 34.2% (1.52/h), as compared to visual scoring. Multiplexed images were superior to scalograms alone (scalogram-kurtosis-entropy 0.956 ± 0.010, scalogram 0.890 ± 0.028, *t*(7) = 3.54, *p* < 0.01) and a DCNN trained specifically for the individual animal was superior to using DCNNs across animals (intra-animal 0.960 ± 0.0094, inter-animal 0.811 ± 0.015,* t*(30) = 5.54, *p* < 0.01). For this dataset the DCNN approach is superior to a previously described algorithm utilizing longer local line lengths, calculated from wavelet-decomposition of EEG, to identify seizures. We demonstrate the novel use of a predesigned DCNN constructed to classify images, utilizing multiplexed images of EEG spectral content, kurtosis, and entropy, to rapidly and objectively identifies seizures in a large dataset of rat EEG with high sensitivity.

## Introduction

Epilepsy is a common neurologic disease characterized by an enduring predisposition to spontaneous seizures. Epilepsy has a prevalence of ~ 1% of the population^1^, and despite extensive efforts in research and clinical care, up to half of people with epilepsy continue to have seizures^2^. These continued seizures result in significant reductions in quality of life^1, 3^ and substantial cost^4^, with direct medical costs related to epilepsy reaching $24.4 billion ($12.5 billion in 1995) per year in the US^5^. Electroencehpalography (EEG) plays a significant role in the diagnosis and quantification of seizures in both clinical care of people with epilepsy and in research studies utilizing animal models of epilepsy. Technological advances allow for collection of vast amounts of EEG data, providing potential benefits albeit with analytical challenges. While EEG is traditionally analyzed by visual inspection aided by quantitative signal analysis tools^6^, this approach is not practical for large datasets both due to efficiency and highly variable inter-observer agreement^7–9^. Therefore, novel methods are needed.

Traumatic brain injury (TBI) is a common cause of epilepsy (post-traumatic epilepsy, PTE), accounting for 10–20% of symptomatic epilepsy^10, 11^. In a rodent model of post-traumatic seizures utilizing a unique inbred strain of rats selected for susceptibility to neuroplasticity (Perforant Path Kindling Susceptible, PPKS rats), frequent spontaneous recurrent seizures are noted in approximately half of injured animals^12^. Using chronic recordings of video-EEG over the course of 5–6 months, extensive collections of EEG data are produced that capture the development and progression of PTE. While classification of animals as having PTE can be easily accomplished by visual review of the recordings at a late time point following TBI, processing the entire dataset by visual inspection is not practical. Conversely only reviewing a subset of the data would result in loss of information and selection bias which may preclude valuable insights.

Machine learning approaches have been used previously to study EEG in both research and clinical settings^13–15^. While several machine learning approaches have been used successfully for human EEG, typically scalp EEG^16–21^, the use of these powerful techniques for rodent EEG is very limited^22^. Feature extraction from EEG typically involves time–frequency spectral content, either using a Fast Fourier Transform (FFT) or Wavelet Transformation (WT). Additional features such as the “sharpness” of waveforms as represented by kurtosis, and spectral entropy, a measure of the information content of a signal that normally decreases during a seizure, can also be extracted. These approaches allow both the ability to summarize large amounts of data visually and to demonstrate patterns which may not be easily recognizable from visual inspection.

These features may be studied using a broad range of machine learning approaches, including convolutional neural networks. Convolutional neural networks have been successful addressing classification in many situations^15^, in part due to the network’s ability to function without human intervention to identify critical features, though these critical features used for classification are not readily extracted from the trained network. Additionally, it has been recognized that pre-existing networks may be repurposed (transfer learning), saving computational effort and avoiding the need for large sets of training data. Specifically, networks designed to discriminate images, such as GoogLeNet, AlexNet, and SqueezeNet, can be used to classify EEG, typically using images of spectral information representing a seizure segment or a baseline, non-seizure segment. GoogLeNet is a 22-layer deep convolutional neural network developed by researchers at Google to solve computer vision tasks including object detection and image classification^23^. GoogLeNet differs from many other deep convolutional neural networks (DCNNs) as it creates a deeper architecture through features including 1 × 1 convolutions in the middle of the architecture and global average pooling.

We hypothesize that a machine learning approach will facilitate seizure identification in a large dataset of EEG collected from rats with PTE. By identification of a large percentage of all seizures from the dataset using an objective, algorithmic method, a more complete understanding of the development and progression of epilepsy in this model will result. This improved understanding is expected to lead to greater insight into the pathological processes underlying post-traumatic epileptogenesis, helping to identify targets for therapeutic intervention and defining biomarkers for evaluating interventions. The use of DCNN with multiplexed images may provide a useful tool for seizure detection and other classification problems.

## Results

### PPKS rats develop PTE following a moderate-to-severe TBI

**Figure 1:**
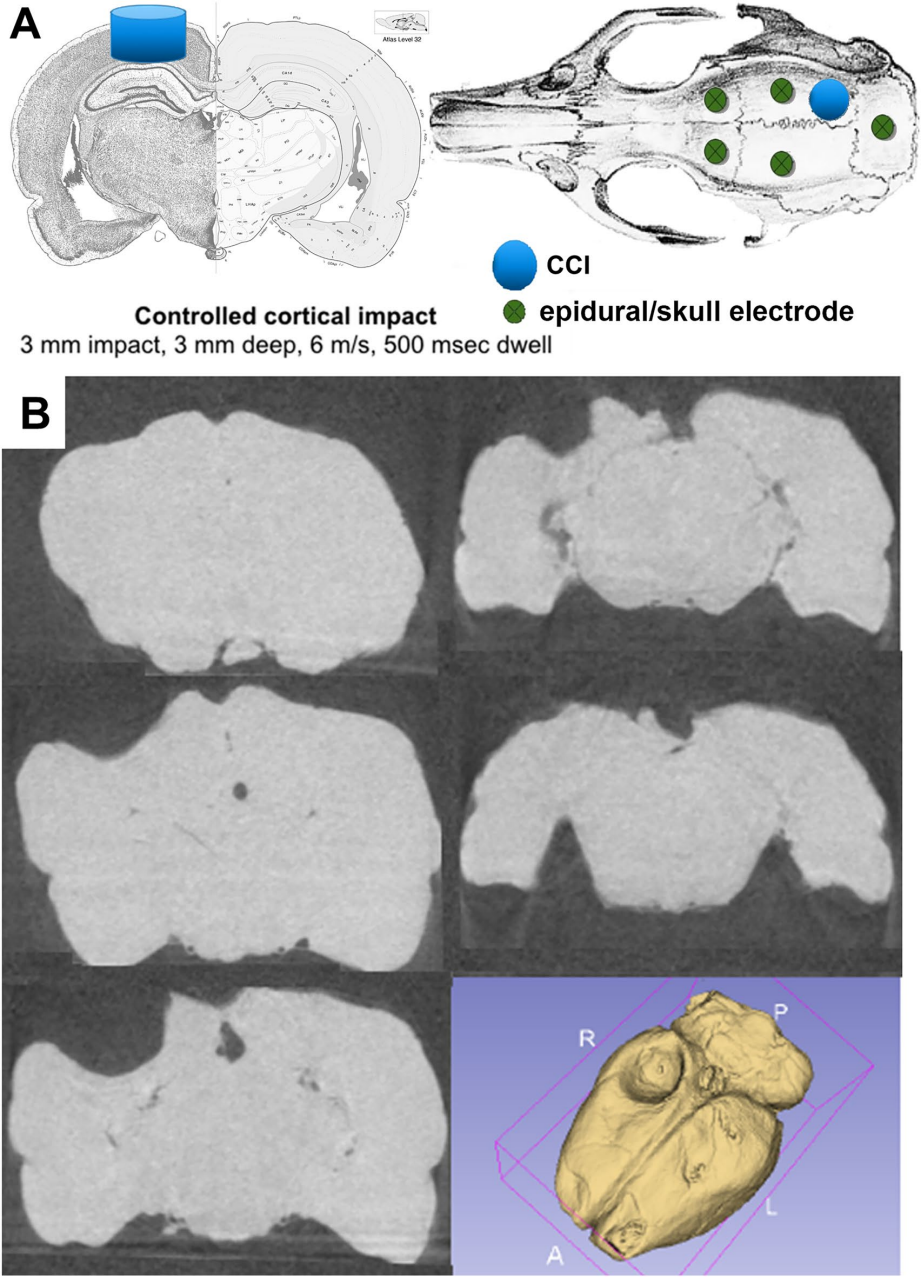
Traumatic brain injury. (**A**) A controlled cortical impact (CCI) is targeted to the posterior cortex, with a depth (3mm) chosen to abut the hippocampus. Epidural electrodes are placed bilaterally anterior and posterior, with a reference electrode in the posterior skull. (**B**) CT imaging with coronal sections and reconstruction demonstrating a representative lesion at 6 months following CCI.

**Figure 2:**
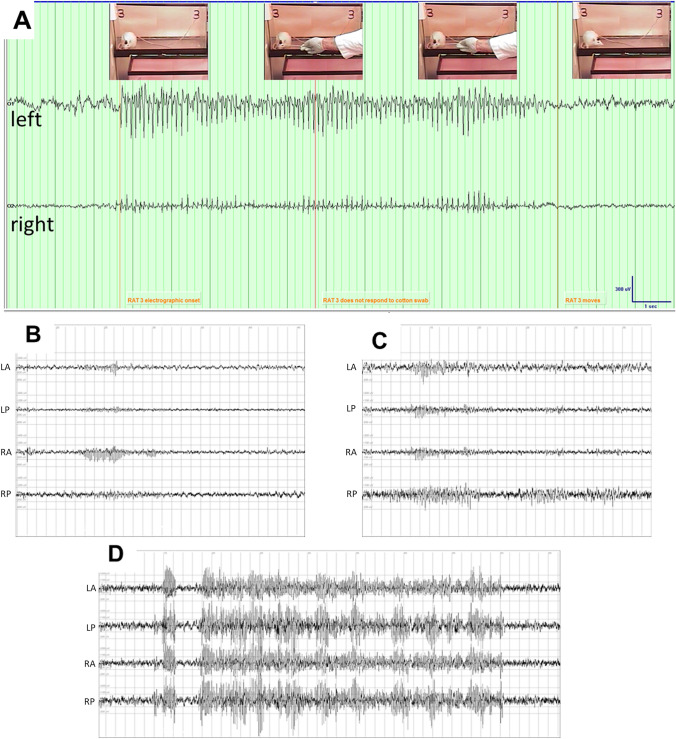
Post-traumatic seizures. (**A**) Seizures seen in rats with PTE typically consist of a behavioral arrest and impaired response to external stimulation (e.g., tapping on cage). Electrographically rhythmic spikes are noted. (**B**) EEG of a focal seizure seen best in the right frontal (RF) electrode. (**C**) EEG of a seizure seen most clearly over the right posterior region, but also in other channels. (**D**) EEG of a seizure seen equally in all channels.

Following TBI (Fig. 1), rats were recorded on average a total of 138 h 13 min (range 118 h 56 min to 166 h 25 min). Spontaneous seizures were detected in 53.3% (16 of 30) of PPKS rats. Seizures were evidenced by rhythmic spikes on the electrographic recording and a clinical correlate of behavioral arrest with subtle oral automatisms and chewing (Fig. 2A). Lack of responsivity to external stimulation (e.g., tapping on glass chamber) during the seizure was documented at least once in each animal demonstrating ictal electrographic activity, confirming that the episode of spike-wave discharges met conventional criteria defining seizures, i.e., abnormal synchronous EEG activity accompanied by clinical impairment consisting of altered responsiveness (Supplementary material video). A range of different electrographic seizure patterns were noted (Fig. 2B–D). Several months after TBI seizures occur frequently (95% CI 10.8–17.5 seizures per hour).

### Generation of a DCNN to classify multiplexed scalograms for seizure identification

**Figure 3:**
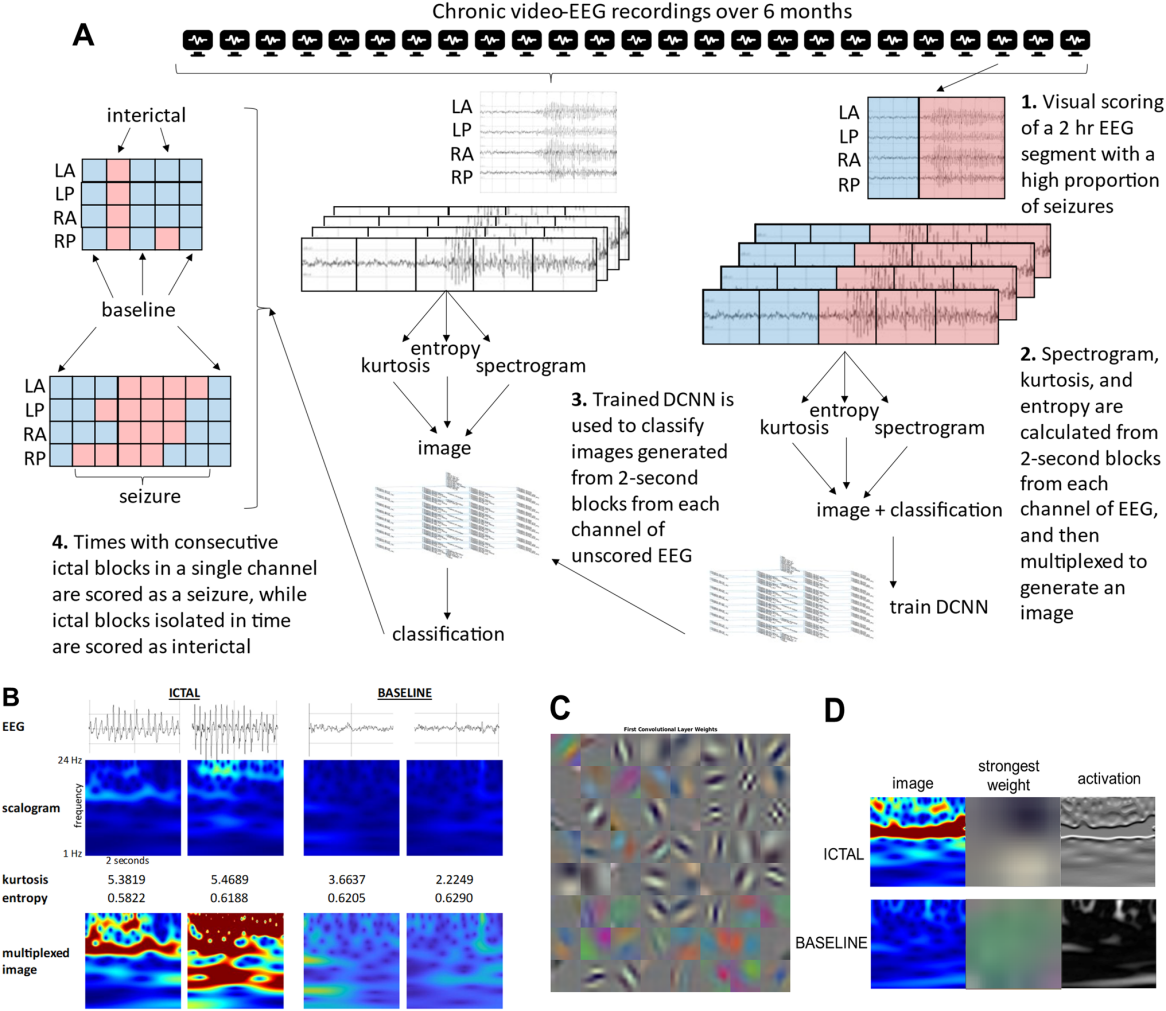
Deep Convolutional Neural Network (DCNN). GoogLeNet is a deep CNN originally designed to classify images into 1000 categories. Each of the 22 layers of the network responds to features of the image, becoming increasing specific with each layer and finally producing a classification. (**A**) Training was performed using a 2-h segment of a recording later (5–6 months) in the recording period, with a high frequency of seizures and good technical quality. The segment was scored by visual inspection and each 2-s block from each channel was classified as ICTAL or BASELINE. For each 2-s block of each channel, a scalogram (1–24 Hz), kurtosis, and entropy are calculated and multiplexed to generate a 224 × 224 × 3 RGB image. Classified images were used for training of the DCNN. The trained DCNN is then used to score multiplexed images from 2-s blocks of other recordings. ICTAL segments isolated in time are scored as interictal, while ICTAL segments with a neighboring ICTAL segment in the same channel are scored as seizures. (**B**) Examples of EEG segments, scalogram, kurtosis, entropy, and multiplexed images. (**C**) Weights of the initial layer respond to low-level features such as edges and colors. (**D**) An example image for ICTAL and BASELINE blocks, with the strongest weight from the layer and resultant activation.

To utilize a machine-learning approach to seizures identification in EEG recordings, we repurposed a predesigned DCNN, GoogLeNet, originally created to classify images^23^ (Fig. 3A). Scalograms were generated from the EEG for each of the four channels, utilizing 2-s segments (Fig. 3B). Scalograms were multiplexed with kurtosis and the inverse of spectral entropy as scalars multiplied to the scalogram and using equal weighting. To support use of these measures in the DCNN, a generalized linear model with a nominal logistic fit examining factors associated with differentiating ICTAL from BASELINE segments revealed significant effects from entropy (β − 46.4, LogWorth 39.080, *p* < 0.01) and kurtosis ((β 0.28, LogWorth 6.359, *p* < 0.01), but not line length ((β − 3.68 × 10^–5^, LogWorth 0.485, *p* = 0.32) (Supplemental material figure). Therefore entropy and kurtosis were multiplexed to the scalogram, while line length was not.

**Figure 4:**
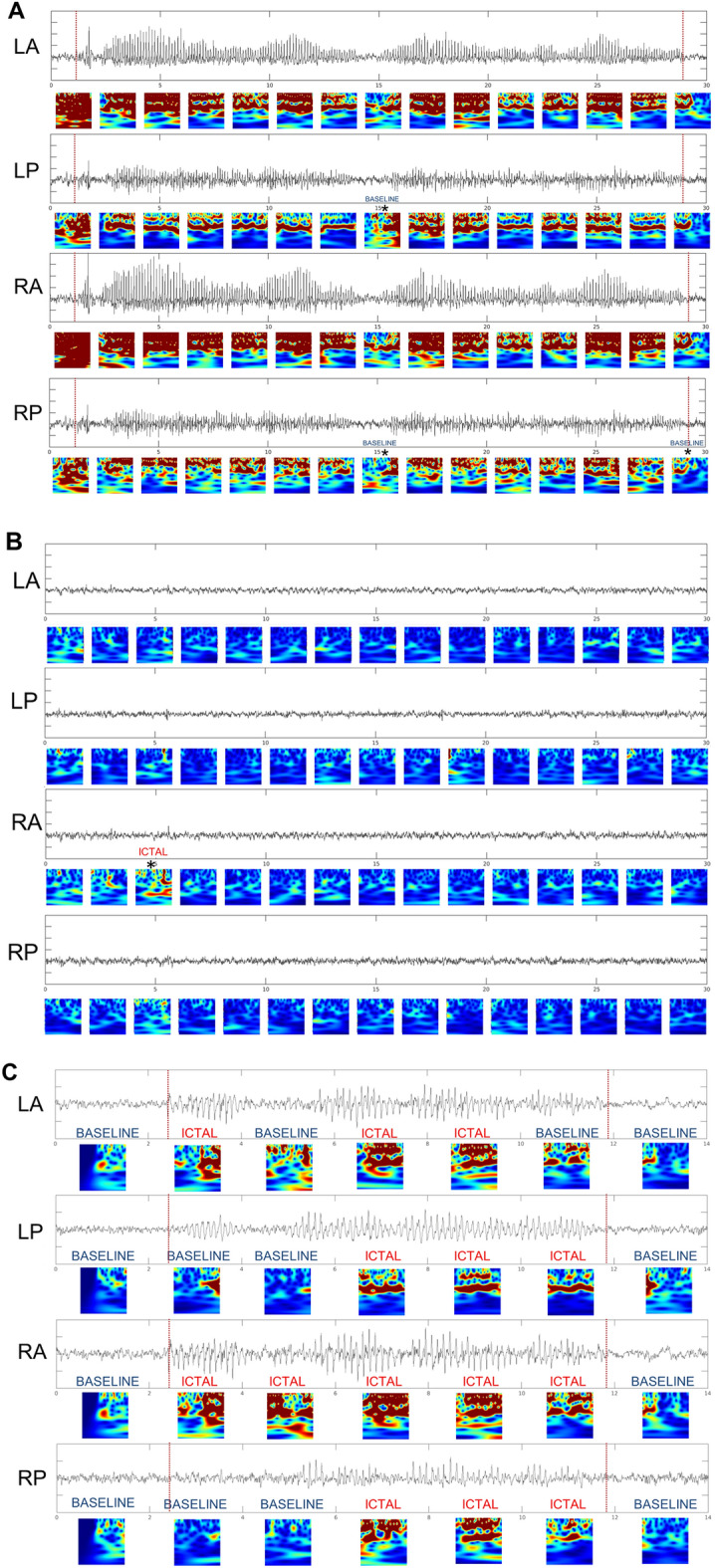
Seizure and baseline recordings. Electrographic recordings from Left Anterior (LA), Left Posterior (LP), Right Anterior (RA), and Right Posterior (RP) and associated multiplexed scalogram images in a PPKS rat that developed recurring seizures after CCI. The horizontal axis is marked in seconds. Seizure beginning and end by visual scoring is marked by vertical dotted red lines in (**A**) and (**B**). (**A**) Example of a generalized spike wave seizure. The trained DCNN identified all except 3 images (marked with *) as ICTAL. (**B**) An epoch of recording from the same animal as in A which demonstrated no spike-wave discharges and was regarded as normal baseline by visual inspection. The trained network identified all except 1 image (marked with *) as BASELINE. (**C**) Example of a generalized spike wave seizure with anterior dominance. Ictal electrographic activity is seen more clearly in some channels (e.g., RA > RP, LA > LP). The trained DCNN identified 14 of 20 images as ICTAL.

For identification of seizures, when the trained DCNN classified ≥ 2 consecutive segments as ICTAL in the same channel (Fig. 3A and RA in Fig. 4C), those time periods were scored as a seizure. Other channels may demonstrate either ICTAL or BASELINE images within that same time periods, allowing for focal seizures to be captured (Figs. 3A, 4C). Isolated segments classified as ICTAL were not scored as a seizure to exclude excessive false positives and isolated interictal epileptiform activity or artifact (Fig. 3A and the third segment for RA in Fig. 4B).

Visual inspection by experienced electroencephalographers was used as the gold standard. Seizure onset and termination by visual inspection are denoted by vertical red dotted lines when applicable (Fig. 4A,C). A 2-s segment was considered ICTAL by visual inspection if more than half (> 1 s) of the segment fell within a seizure marked by visual inspection. Only events with ictal electrographic activity lasting ≥ 4 s were marked by visual inspection. For seizure detection accuracy, a DCNN seizure detection overlapping with a seizure marked by visual inspection was considered a true positive.

### Multiplexed scalograms including kurtosis and spectral entropy produce superior accuracy to scalograms alone

**Figure 5:**
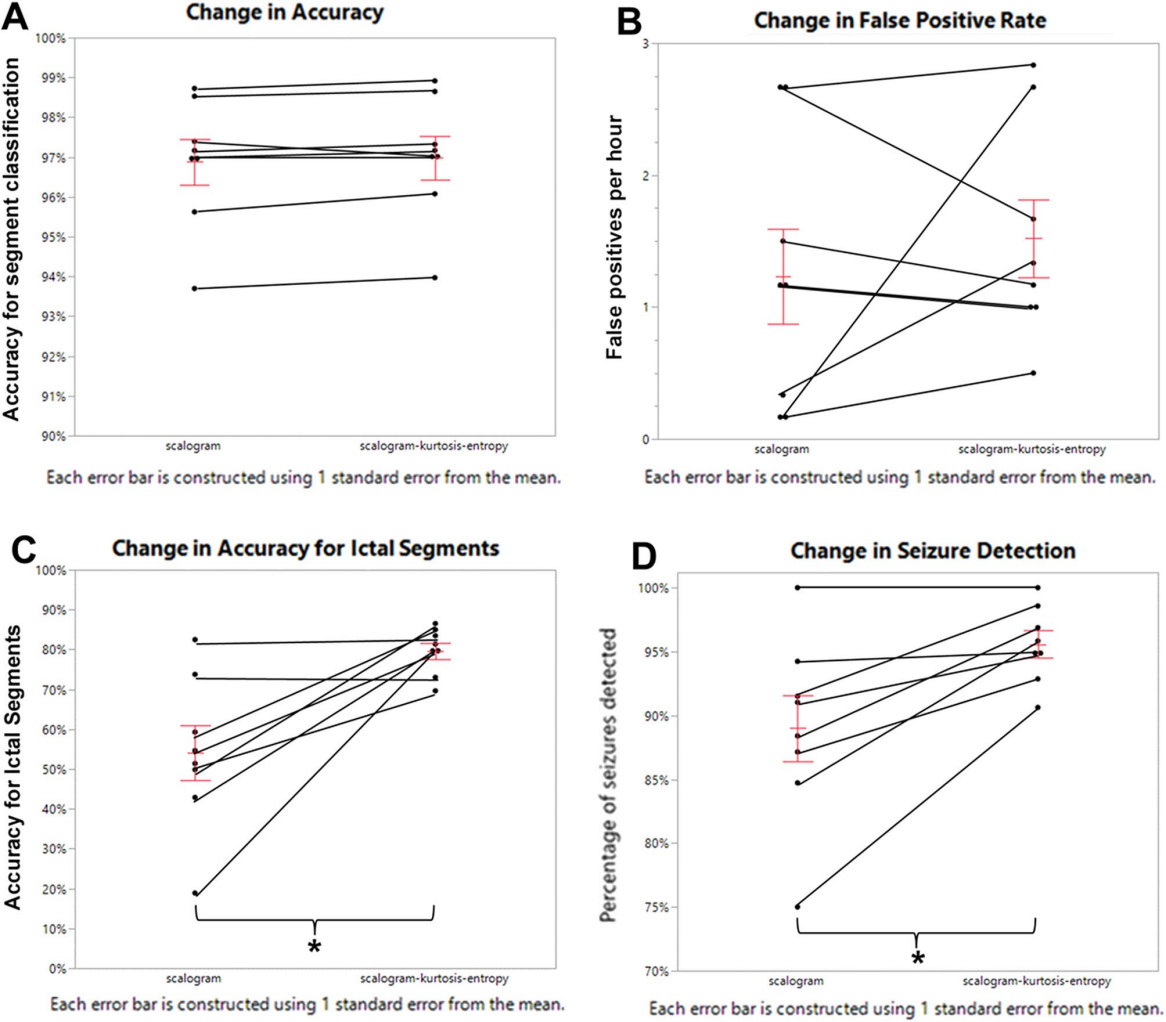
Performance of Multiplexed scalogram versus scalogram alone. (**A**) Using multiplexed scalograms, scaled by kurtosis and the inverse of entropy, did not significantly alter overall accuracy of the DCNN for both ICTAL and BASELINE blocks (scalogram 0.969, scalogram-kurtosis-entropy 0.970, *p* > 0.05). (**B**) The false positive rate did not significantly differ between scalograms and multiplexed scalograms (scalogram 1.23 per hour, scalogram-kurtosis-entropy 1.52 per hour, *p* > 0.05). (**C**) Multiplexed scalograms resulted in a significant improvement in ICTAL segments (scalogram 0.541, scalogram-kurtosis-entropy 0.797, *p* < 0.01). (**D**) Multiplexed scalograms resulted in a significant improvement in detection of seizures (scalogram 0.890, scalogram-kurtosis-entropy 0.956, *p* < 0.01).

Comparison of DCNNs trained on multiplexed scalograms including kurtosis and spectral entropy (scalogram-kurtosis-entropy) to scalograms alone demonstrated superiority for the multiplexed images, most importantly in the accuracy for seizure detection. For a subset of animals (*n* = 8), the network was trained from a 2-h segment of a recording with high frequency of seizures (1–10% of recording) and of good technical quality, using either images constructed from the scalogram alone or the scalogram-kurtosis-entropy multiplexed image. The trained DCNN was then used to classify the remaining 6 h of the same EEG record. No statistically significant difference was found in overall accuracy compared to scoring by visual inspection between the two training sets (scalogram 0.969 ± 0.0061, scalogram-kurtosis-entropy 0.970 ± 0.0058, *t*(7) = 1.14, *p* > 0.05) (Fig. 5A). Similarly, the rate of false positives did not differ between DCNNs trained using images constructed from the scalogram alone or the scalogram-kurtosis-entropy multiplexed image (scalogram 1.23 ± 0.38/h, scalogram-kurtosis-entropy 1.52 ± 0.31/h, *t*(7) = 0.778, *p* > 0.05) (Fig. 5B). However, comparison of accuracy for ICTAL segments (percentage of images classified as ICTAL within a seizure detection) was significantly improved by use of multiplexed scalogram-kurtosis-entropy images (scalogram 0.541 ± 0.073, scalogram-kurtosis-entropy 0.797 ± 0.022, *t*(7) = 3.58, *p* < 0.01) (Fig. 5C). Similarly multiplexed scalogram-kurtosis-entropy images resulted in a significant improvement in detection of seizures as compared to images from scalograms alone (scalogram 0.890 ± 0.028, scalogram-kurtosis-entropy 0.956 ± 0.010, *t*(7) = 3.54, *p* < 0.01) (Fig. 5D).

A limited exploration of the effect of changing the weights used for kurtosis and inverse of entropy was performed (*n* = 5), training the DCNN on a 2-h segment of a recording with a high frequency of seizures and evaluating the trained model on the remaining 6 h of the same recording, as above. For this group, using the standard equal weights for kurtosis and entropy, ICTAL segments were correctly identified with an accuracy of 0.785 ± 0.0335. Including only kurtosis (kurtosis weight 1 and entropy weight 0) resulted in a significant decrease in accuracy (0.657 ± 0.0626, *p* < 0.05). A strong trend toward a decrease in accuracy was seen when including only entropy (kurtosis weight 0 and entropy weight 1) (0.676 ± 0.0482, *p* = 0.06) and weaker trends toward a decrease in accuracy when kurtosis is weighted 5:1 over entropy (0.704 ± 0.103, *p* = 0.21) or entropy is weighted 5:1 over kurtosis (0.712 ± 0.0676, *p* = 0.28).

### DCNN trained for each animal (intra-animal) is superior to DCNN used across animals (inter-animal)

Comparison of DCNNs trained on a single animal and used for evaluation of recordings for that same animal (intra-animal) to DCNNs trained on the recordings of one animal and used to classify other animals (inter-animal) demonstrated superior accuracy for seizure detection with intra-animal trained networks. For a subset of animals (*n* = 8), the network was trained from a 2-h segment of a recording with high frequency of seizures (1–10% of recording) and good technical quality using scalogram-kurtosis-entropy images. The trained network was then used to classify images from additional recordings, either from the same animal (intra-animal) or from a different animal (inter-animal).

**Figure 6:**
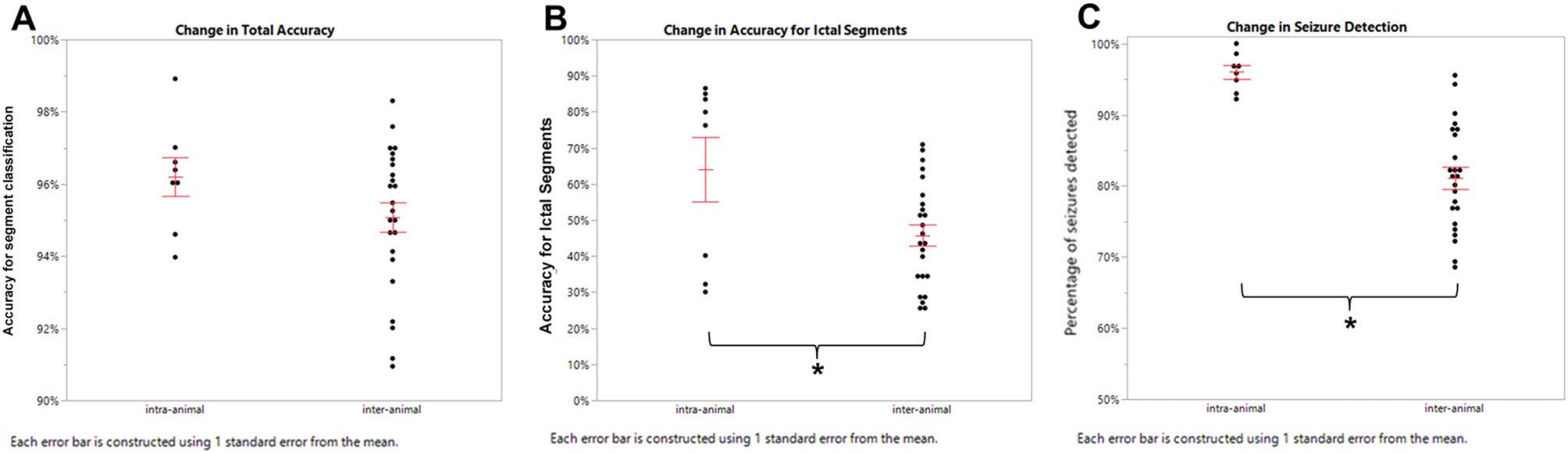
Comparison of intra-animal and inter-animal networks. (**A**) The difference in overall accuracy between a DCNN trained on the same animal (intra-animal) but used across recordings at other times and DCNN trained on a different animal (inter-animal) was not statistically significant (intra-animal 0.962, inter-animal 0.951, *p* > 0.05). (**B**) Intra-animal networks had greater accuracy for ICTAL segments than inter-animal networks (intra-animal 0.641, inter-animal 0.478, *p* < 0.05). (**C**) Intra-animal networks had greater accuracy for seizure detection than inter-animal networks (intra-animal 0.960, inter-animal 0.811, *p* < 0.01).

The difference in overall accuracy between a DCNN trained on the same animal used at different timepoints, and a DCNN trained on one animal and used to classify the recording of a different animal was not statistically significant (intra-animal 0.962 ± 0.0053, inter-animal 0.951 ± 0.0041,* t*(30) = 1.46, *p* > 0.05) (Fig. 6A). However, when comparing accuracy for ICTAL segments, intra-animal networks had greater accuracy than inter-animal networks (intra-animal 0.641 ± 0.089, inter-animal 0.478 ± 0.029, *t*(30) = 2.56, *p* < 0.05) (Fig. 6B). Most importantly, for seizure detection intra-animal networks also had greater accuracy than inter-animal networks (intra-animal 0.960 ± 0.0094, inter-animal 0.811 ± 0.015, *t*(30) = 5.54, *p* < 0.01) (Fig. 6C).

### DCNN demonstrates superior accuracy and false positive rate as compared to a wavelet decomposition algorithm

**Figure 7:**
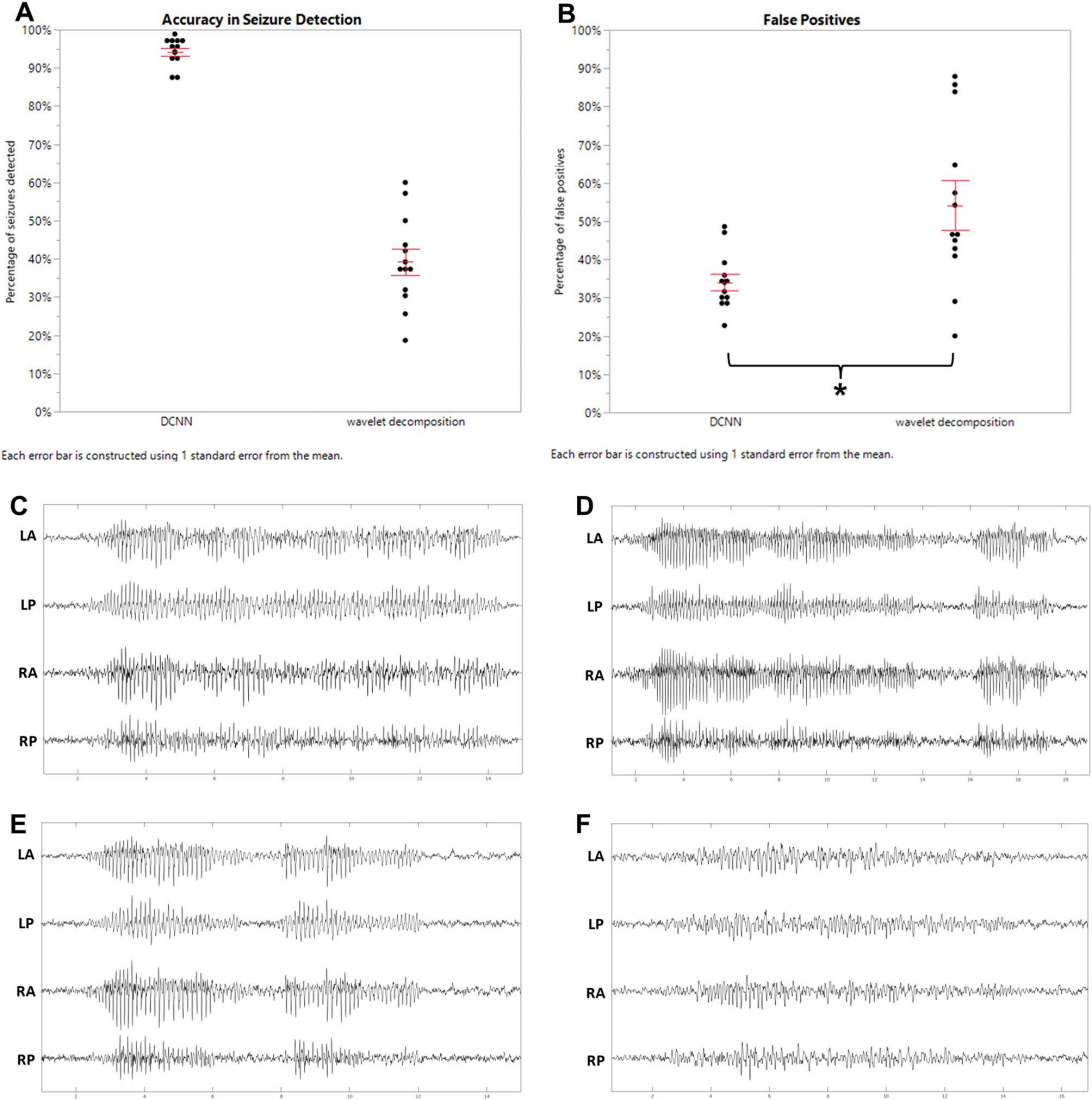
Comparison of DCNN and wavelet decomposition approaches. (**A**) The DCNN identified a significantly higher percentage of seizures as compared to the wavelet decomposition algorithm (DCNN 0.942, wavelet decomposition 0.392, *t*(11) = 13.79, *p* < 0.01). (**B**) The false positive rate was found to be significantly lower for the DCNN than for the wavelet decomposition algorithm (DCNN 0.342, wavelet decomposition 0.542, *t*(11) = 3.21, *p* < 0.01). (**C**–**E**) Examples of false negatives from the wavelet decomposition algorithm identified by the DCNN. (**F**) Example of false negatives of the DCNN identified by the wavelet decomposition algorithm.

Using a subset of recordings from the later period (5–6 months after TBI) in which seizures were more frequent, a DCNN trained with multiplexed images and used intra-animal was compared to an algorithm which utilized total variation in line length following wavelet decomposition, previously developed by Bergstrom and colleagues^22^. The wavelet decomposition algorithm utilized a baseline period without seizures, selected from the same recording that was scored by the algorithm. Wavelet decomposition levels of 2–5 and window lengths of 125 ms, 250 ms, and 500 ms were examined, and the parameters with the highest accuracy for seizure detection used for comparison to the DCNN. In comparison to visual scoring the DCNN identified 94.2 ± 1.1% of seizures, as compared to 39.2 ± 3.5% for the wavelet decomposition algorithm (*t*(11) = 13.79, *p* < 0.01) (Fig. 7A). For the same dataset the false positive rate was found to be 34.2 ± 2.2% for the DCNN and 54.2 ± 6.4% for the wavelet decomposition algorithm (*t*(11) = 3.21, *p* < 0.01) (Fig. 7B).

## Discussion

We hypothesized that a machine learning approach would be able to facilitate seizure identification in a large dataset of EEG recorded from rats. We demonstrated that (1) a predesigned DCNN (GoogLeNet) could be trained to detect seizures by classifying images representing statistical features of the EEG, (2) multiplexed images combining information from the scalogram, kurtosis, and spectral entropy produced superior results as compared to images generated from the scalogram alone, (3) using a DCNN within an animal was superior to using a DCNN across animals, and (4) the DCNN was superior in percentage of seizures detected and in rate of false positives as compared to an algorithm utilizing total variation in line length following wavelet decomposition^22^. We believe that this novel method will advance the analysis of large electrographic datasets.

While identification of rats with post-traumatic seizures is achievable by the gold standard of visual inspection of the EEG, given the extensive duration of video-EEG recordings (2211 h 33 min total for all 16 rats with post-traumatic seizures) and high frequency of seizures at later times following TBI, identification of a high percentage of all the seizures is not easily achievable. Furthermore, identifying seizures by visual inspection involves considerable subjectivity^7–9, 24, 25^ which may introduce bias into analysis. Therefore, an algorithmic approach was sought. A machine learning approach to identify seizures can avoid both these issues through its ability to rapidly process large amounts of data and to identify seizures in an objective, algorithmic manner. We have demonstrated that a predesigned DCNN (GoogLeNet) can be trained using images representing multiplexed statistical features of the EEG to identify seizures and validated this approach by comparison to seizure identification by conventional visual inspection. Using this approach, we were able to identify a high percentage (~ 95%) of all seizures from a dataset of more than 2000 h of EEG.

We demonstrated that multiplexing kurtosis and spectral entropy with the scalogram significantly improved the identification of ICTAL images and in the detection of seizures, without a significant increase in the rate of false positives. The incorporation of these scalar features into training images of the DCNN suggests that a similar multiplexing approach could be useful in other situations in which both images and scalar features may be useful for classification by a machine-learning approach. Furthermore, the weighting of the scalars, provides an additional level of tuning for the model, and a limited evaluation of relative weighting suggested equal weighting of kurtosis and inverse of spectral entropy performed better than using a single scalar or over-weighting one by 5 over the other.

While various measures are taken to generate uniform EEG recordings in humans and animals, several difficult-to-control factors can alter the recorded EEGs. Most importantly in these experiments, even using CCI which is produces a reproducible impact to the brain, the resultant brain injury and subsequent seizures in a subset of animals develop as a cascade of a plethora of mechanisms, each with individual variability. Therefore, it is unsurprising that the electrographic correlate of the seizures is subtly different for each animal. Furthermore, the epidural electrodes likely vary slightly in electrical resistance and location, which while inconsequential for visual inspection produces differences which impact quantitative analysis. While using a DCNN across animals produced fair accuracy for the detection of seizures (81.1 ± 1.3%) and would be expected to be adequate for identification of animals with post-traumatic seizures, using a DCNN trained for the specific animal resulted in a statistically significant improvement in accuracy for seizure detection (96.0 ± 2.3%, *p* < 0.01).

Machine learning and other algorithmic approaches to detecting seizures in large EEG datasets has been used previously, the majority of these approaches have been designed for human EEG^16–21^, typically acquired in a clinical setting. The analysis of EEG acquired from rodent models of epilepsy differs in several important respects, including fewer channels, lack of a standardized placement of electrodes, and greater artifact. The presented methodology produces superior to an algorithmic approach utilizing total variability following wavelet decomposition (Fig. 7). This finding may have been expected as this algorithm utilizes line length and line length was not found to be a significant predictor for seizures in this dataset (Supplemental material figure). Another approach utilized a Random Forest approach in a model of post-traumatic seizures had an accuracy of 91%, sensitivity of 97%, and specificity of 87%^26^, though detailed methods for this approach are not available to allow for direct comparison. Notably both of these approaches required intra-animal adjustments. DCNN approaches have been used for the detection of seizures in human EEGs, generally using feature extraction rather than raw EEG and most commonly frequency distribution^27^. Accuracy for detection of brain states from the EEG, often including pre-ictal states for seizure prediction, is typically > 90%^21, 27, 28^.

Several limitations impact the current work. A wide variety of machine learning approaches could be taken and even for predesigned DCNNs developed for image classification many alternatives are available (e.g., AlexNet and SqueezeNet). Furthermore, many signal analysis techniques are available to summarize the EEG and many methods could be used for multiplexing. Therefore achieving the optimal approach is not easily accomplished. Additionally, while most seizures are unambiguous on EEG, an ictal-interictal spectrum exists and any cut-off for the definition of seizure is necessarily arbitrary. Many algorithms and computations approaches based on analysis of waveform morphology are limited by the frequent occurrence of artifacts in EEG recordings. Some of these challenges are obviated by use of a DCNN trained to detect seizures based on validated conventional ictal EEG patterns. Finally, while training and using the DCNN within an animal was feasible for the goals of the current study, it would limit use in cases in which it is not known if the animal has seizures or in which the frequency of seizures was low enough to preclude identification of a training set. Efforts towards pre-processing of EEG recordings to produce greater uniformity are underway.

In conclusion, our results demonstrate an effective and efficient machine learning approach to the identification of seizures from a large dataset of EEG recorded from freely behaving rats. A high proportion (> 95%) of all seizures from a large data set of EEG spanning multiple animals over months were identified. While few algorithmic approaches have been presented in sufficient detail to allow reproduction, the DCNN demonstrates superior to an approach utilizing total line length variability following wavelet decomposition^22^. Collection of this data in an objective, algorithmic fashion avoids biases associated with identification by visual inspection and loss of information inherent in reviewing only a subset of the data. This approach allows for a more comprehensive study of this animal model and facilitates efforts to understand processes such as progression in epilepsy. Given the dearth of such tools for analysis of rodent EEG, this machine learning approach is an important addition. As technologies increasingly allow for collection of large amounts of electrographic data in both research and clinical settings, new methods are needed to process and summarize information. As these problems are not unique to electrographic recordings, solutions generated for other circumstances, such as image classification, can be repurposed, as this approach demonstrates.

## Materials and methods

The novel Perforant Path Kindling Susceptible (PPKS) strain of rats used in this study were bred (> 15 generations) from a colony at the University of Wisconsin-Madison^29^. Animals were maintained under 12 h light: 12 h dark cycles, with ad libitum food and water, in a vivarium under the care of the University of Wisconsin-Madison veterinarians. All animal handling and procedures were performed according to the NIH Guide for the Care and Use of Laboratory Animals and the experiments were conducted under a protocol approved by the University of Wisconsin Institutional Animal Care and Use Committee. The study is reported in accordance with ARRIVE guidelines.

### Surgical procedure

Rats were 3–4 months of age at the time of surgery. Surgical anesthesia was achieved with isoflurane, with brief induction at 5% and then maintenance at 1–5% (monitored by assessment of withdrawal to paw pinch and corneal reflexes), delivered in 100% oxygen. The rats received atropine (0.02–0.05 mg/kg IM) to reduce respiratory secretions and bupivacaine subcutaneously to pressure points for the stereotaxic stage (external ears bilaterally), at the site of the surgical incision at the midline scalp, and spritzed on the skull prior to drilling. After achieving surgical anesthesia, the hair was shaved over the operative site and the skin was cleaned with povidone-iodine. An incision was made in the scalp to expose the cranial surface and bleeding was controlled by electrocautery.

### Controlled cortical impact (CCI)

Isoflurane was maintained at 1–2% for at least 5 min prior to delivery o the CCI. The craniotomy was made over the right posterior quadrant with the dura left intact (Fig. 1A) with a sterile, circular 5 mm diameter trephine. A Leica Impact One Stereotaxic Impactor delivered the CCI with a 3 mm diameter impactor, blunt tipped. CCI is delivered to a depth of 3 mm, with an impact velocity of 6 m/s and a dwell time of 500 ms, targeting the posterior cortex and extending through cortex but not directly into hippocampus (Fig. 1A). The craniotomy was then covered by Gelfoam (Pfizer).

### Electrode implantation

Following the CCI, four burr holes were drilled into the epidural space in each quadrant, and screw electrodes were placed into the burr holes, as well as in a blind hole into the skull overlying the cerebellum used as a ground and reference electrode (Fig. 1A). Following placement of the electrodes, hemostasis was achieved and the electrodes attached to the skull by a dental acrylic cap. The remaining scalp wound was closed, as needed, with suture (Ethicon 4-0 dissolvable chromic gut). The surgical site was treated with triple antibiotic ointment. The usual duration of surgery was 20–30 min. With the exclusive use of isoflurane inhalation anesthesia, rats typically resumed ambulation within minutes of returning to normal ambient air. While recovering, all rats were warmed with an incandescent lamp. The lamp was kept 8″ or more above and at the edge of the cage to prevent burns. Postoperative analgesia was administered, with rats receiving flunixin (2 mg/kg IM daily) immediately following the surgical procedure and once daily for the first three post-operative days. Rats were followed for signs of pain such as weight loss, poor grooming, or decreased movement.

### Video-EEG recording

In vivo neurophysiological recordings were conducted on freely behaving rats. The rats were connected by a recording cable through the chronic headstage to record spontaneous EEG and animals were housed in 10″ × 12″ × 22″ glass chambers to allow recording of simultaneous video. Video-EEG was recorded using an XLTEK EEG system (Neuroworks, 8.5.1) with a Connex Brain Monitor amplifier (sampled at 1024 Hz). Four channels of EEG were recorded, labeled Left Anterior (LA), Left Posterior (LP), Right Anterior (RA), and Right Posterior (RP), with each of the epidural electrodes referenced to the electrode placed in a blind hole in the posterior skull (Fig. 1A). The recording sessions lasted 4–8 h and occurred 1–2 times per week and were directly observed by laboratory staff. At the conclusion of the recording period rats were returned to their regular housing. Video-EEG recordings began 1–2 weeks after CCI and continued for 5–6 months. Video-EEG recordings were monitored for quality control and preliminarily screened for the presence of seizures during the chronic recording period.

Visual inspection of the recorded EEG was initially used to identify rats experiencing post-CCI seizures defined by conventional criteria for seizure classification including trains of rhythmic generalized spike-waves, focal spike/spike-wave discharges, and focal spike/spike-wave with secondary generalization (Fig. 2). Isolated spikes, sharp waves, and other isolated interictal patterns were not considered as evidence of posttraumatic seizures. At the end of the recording period (5–6 months after CCI) rats were definitively classified as having or not having post-traumatic seizures based on visual review of the video-EEG recordings.

Video-EEG recordings were scored for seizures by visual inspection through a tiered approach in which reviewers with a basic level of training and experience performed the primary scoring. The primary scoring was assessed by a reviewer with an advance level of training and experience, and the second-level scoring was reviewed by an experienced epileptologist (RJK, TPS, PAR).

### Classification of EEG by deep convolutional neural network (DCNN)

To objectively and rapidly analyze the large EEG dataset, an approach utilizing a predesigned deep convolutional neural network (DCNN) devised to classify images (GoogLeNet) was chosen to identify seizures (Fig. 3A). For each rat with post-traumatic seizures as identified and classified by visual review described above, a recording of 2 h duration and good technical quality near the end of the recording period (at least 5 months since TBI) was chosen for training of the DCNN. Recordings at this time interval after CCI in the subset of rats which developed seizures typically demonstrated a high frequency of seizures. The duration of episodes of ictal activity was determined by visual inspection, with the onset marked as the earliest spike initiating a rhythmic train of spike discharges, in any of the 4 channels, and cessation of ictal activity identified when spike discharges were not noted in any channel. Post-ictal electrographic slowing was not scored as seizure. The entire recording was divided into 2-s segments and a segment was classified as ICTAL if > 1 s was marked as seizure by visual inspection, else the segment was classified as BASELINE.

Analysis was performed in MATLAB (R2021a) and was developed from a MATLAB example^30^. For each 2-s segment of each channel continuous wavelet transformation was used to generate a scalogram from 1 to 24 Hz (12 voices per octave) to produce a 224 × 224 × 3 RBG image (scalogram). The scalogram was multiplied by the kurtosis and the inverse of spectral entropy, each calculated for the 2-s segment, to produce a 224 × 224 × 3 multiplexed image (scalogram-kurtosis-entropy) (Fig. 3B).

Training the DCNN was performed utilizing a 2-h segment of EEG with a high frequency of seizures (1–10% of recording), with the remainder of the recording used as validation. GoogLeNet (Deep Learning Toolbox, MATLAB) was chosen as the predesigned network (Fig. 3C). Training utilized a batch size of 15, a maximum of 20 epochs, and an initial learning rate of 0.0001^30^. The trained network was then used to classify images from additional recordings as ICTAL or BASELINE (Fig. 3D). For seizure detection, a 2-s segment was classified as a seizure when two or more consecutive ICTAL segments were identified in a channel, while isolated 2-s classified as ICTAL were scored as interictal activity. Seizures identified by the DCNN were reviewed by visual inspection to exclude false positives.

### Statistical analysis

All results are presented as mean ± SEM. All statistical tests were performed by JMP Pro 15 (SAS Institute, Inc). A generalized linear model utilizing a nominal logistic fit for kurtosis, entropy, and line length for either ICTAL or BASELINE 2-s EEG blocks. Accuracy for the DCNN was compared to scoring by visual inspection. Paired t-tests were utilized for comparison of training utilizing the scalogram images or the multiplexed scalogram-kurtosis-entropy images. The exploration of weights for kurtosis and entropy was analyzed by an ANOVA with post-hoc analysis by Dunnett’s method with equal weights as the control. A pooled t-test was utilized to compare accuracy of the DCNN trained and applied within a single animal (intra-animal) or trained and applied across animals (inter-animal).

### Supplementary Information

Supplementary Legends.

Supplementary Video 1.

Supplementary Information 1.

Supplementary Information 2.

## Data Availability

All data related to the DCNN and its application are included in this published article (and its Supplementary material). The EEG datasets analysed during the current study are not publicly available due to ongoing analysis and subsequent publications but are available from the corresponding author on reasonable request.

## Abbreviations

CCI: Controlled cortical impact
DCNN: Deep convoluted neural network
TBI: Traumatic brain injury
