# Supplementary material for: A machine learning approach to seizure detection in a rat model of post-traumatic epilepsy
Source: Sci Rep. 2023 Sep 22;13:15807. doi: 10.1038/s41598-023-40628-1 (PMC10517002; doi:10.1038/s41598-023-40628-1)
Supplement: Supplementary file 1 — Supplementary Legends. [file 41598_2023_40628_MOESM1_ESM.docx]

# Supplementary figure legends

**Supplementary material 1-video. Post-traumatic seizure video-EEG.** Given the high frequency of seizures in this model of post-traumatic epilepsy, responsiveness to stimulation can be tested. A video-EEG demonstrates a rat in the recording cage. EEG demonstrates the onset of the electrographic correlate of a seizure with rhythmic polyspikes seen over the left (top channel) and right (bottom channel) hemispheres, lasting 11 seconds. The rat stops moving and does not respond to tapping on the glass cage during the seizure (behavioral arrest, unresponsive), but begins moving again after the seizure ends. This seizure is presented as an image in Figure 2A.

**Supplementary material 2-figure. Multiplexed scalars.** The parameter estimates from the GLM for kurtosis, entropy, and line length generated in JMP. Definitions and equations for MATLAB functions pentropy and kurtosis.

**Supplementary material 3-matlab_code. MATLAB code.** MATLAB code for generating training images for initial training of the DCNN, training the DCNN, and classifying images from unscored EEG recording with the DCNN.
