# Supplementary material for: A machine learning approach to seizure detection in a rat model of post-traumatic epilepsy
Source: Sci Rep. 2023 Sep 22;13:15807. doi: 10.1038/s41598-023-40628-1 (PMC10517002; doi:10.1038/s41598-023-40628-1)
Supplement: Supplementary file 3 — Supplementary Information 1. [file 41598_2023_40628_MOESM3_ESM.docx]

**Supplemental Figure #2**

**GLM Parameter Estimates**


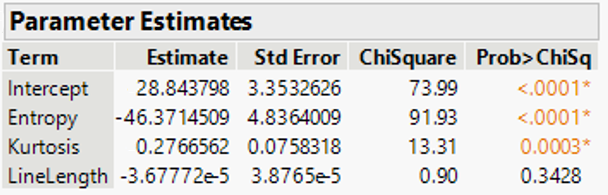


The MATLAB functions pentropy[1] and kurtosis[2] are defined as:

For a signal x(n), the power spectrum is S(m) = |X(m)|^2^, where X(m) is the discrete Fourier transform of x(n). The probability distribution P(m) is then:


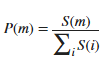


The spectral entropy H follows as:


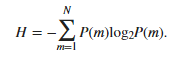


The kurtosis of a distribution is defined as


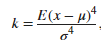


where *μ* is the mean of *x*, *σ* is the standard deviation of *x*, and *E(t)* represents the expected value of the quantity *t*.

**References:**

1. Spectral entropy of signal - MATLAB pentropy. https://www.mathworks.com/help/signal/ref/pentropy.html. Accessed 8 Aug 2023

2. Kurtosis - MATLAB kurtosis. https://www.mathworks.com/help/stats/kurtosis.html. Accessed 8 Aug 2023
